# First identification and characterization of ovine gammaherpesvirus type 2 in horses and artiodactyla from an outbreak of malignant catarrhal fever in Mexico

**DOI:** 10.1371/journal.pone.0290309

**Published:** 2023-09-01

**Authors:** Tania Lucia Madrigal-Valencia, Manuel Saavedra-Montañez, Armando Pérez-Torres, Jesús Hernández, Joaquim Segalés, Yesmín Domínguez Hernández, Irma Eugenia Candanosa-Aranda, Alfredo Pérez-Guiot, Humberto Ramírez-Mendoza

**Affiliations:** 1 Departamento de Microbiología e Inmunología, Facultad de Medicina Veterinaria y Zootecnia, UNAM, Mexico City, Mexico; 2 Departamento de Biología Celular y Tisular, Facultad de Medicina, Universidad Nacional Autónoma de México (UNAM), Mexico City, Mexico; 3 Laboratorio de Inmunología, Centro de Investigación en Alimentación y Desarrollo, A.C. (CIAD), Hermosillo, Sonora, Mexico; 4 Unitat Mixta d’Investigació IRTA-UAB en Sanitat Animal, Centre de Recerca en Sanitat Animal (CReSA), Campus de la Universitat Autònoma de Barcelona (UAB), Bellaterra, Barcelona, Catalonia, España; 5 Department de Sanitat i Anatomia Animals, Facultat de Veterinària, Campus de la Universitat Autònoma de Barcelona (UAB), Bellaterra, Barcelona, Catalonia, España; 6 Centro de Enseñanza, investigación y Extensión en Producción Animal en Altiplano (CEIEPAA), Facultad de Medicina Veterinaria y Zootecnia (FMVZ), Universidad Nacional Autónoma de México (UNAM), Tequisquiapan, Queretaro, Mexico; 7 División de Ciencias de la Vida, Campus Irapuato-Salamanca, Universidad de Guanajuato, ExHda El Copal, Irapuato, Guanajuato, Mexico; Beni Suef University Faculty of Veterinary Medicine, EGYPT

## Abstract

Ovine gammaherpesvirus 2 (OvHV-2), a member of the genus Macavirus, causes sheep-associated malignant catarrhal fever (SA-MCF), a fatal lymphoproliferative disease affecting a wide variety of ungulates in addition to horses. This study described an outbreak of SA-MCF in Mexico and the identification of the OvHV-2 virus in primary rabbit testis cultures through the generation of intranuclear inclusion bodies, syncytia, immunofluorescence (IF), immunocytochemistry (ICC), immunohistochemistry (IHC), endpoint polymerase chain reaction (PCR), and partial sequencing of the *ORF75* gene. The animals involved in this outbreak showed mucogingival ulcers in the vestibule of the mouth and tongue, hypersalivation, corneal opacity, reduced food consumption, and weight loss of variable severity. These clinical signs and the histopathological findings suggested the diagnosis of SA-MCF. Buffy coat fractions from the anticoagulated blood samples of ill animals were collected and analyzed by PCR. Positive buffy coats were used to inoculate the primary cell cultures of rabbit testis to identify the virus. Small clusters of refractile cytomegalic cells, characteristic of viral cytopathic effects, were observed between 48 and 72 h post-infection. Furthermore, intranuclear acidophilic inclusion bodies (IBs) were identified in the inoculated primary culture cells, and the cytoplasm showed immunoreactivity with hyperimmune rabbit serum against OvHV-2. Moreover, in the liver histological sections from sick deer, immunoreactive juxtanuclear IBs were identified with the same rabbit hyperimmune serum. The obtained sequences were aligned with the OvHV-2 sequences reported in GenBank and revealed a nucleotide identity higher than 98%. Based on the evidence provided in this study, we conclude that the outbreak of SA-MCF in the municipality of Tequisquiapan in the state of Queretaro, Mexico, was caused by OvHV-2. This is the second study reporting that horses are susceptible to OvHV-2 infection and can develop SA-MCF. We identified for the first time in Mexico, the presence of OvHV-2 in buffy coats from horses and Artiodactyla.

## Introduction

Malignant catarrhal fever (MCF) is a severe lymphoproliferative disease in susceptible ungulate species of the Artiodactyla order. MCF can be caused by five viral species of the genus Macavirus: ovine herpesvirus-2 (OvHV-2), alcelaphine herpesvirus-1 (AlHV-1), alcelaphine herpesvirus 2 (AlHV-2), caprine herpesvirus 2 (CpHV-2), and an MCF virus (MCFV) in white-tailed deer (*Odocoileus virginianus*) for which domestic goats can be a carrier host [1].

The two most well-studied are OvHV-2 and AlHV-1, and their infections are asymptomatic in sheep and wildebeest reservoir populations, respectively [2]. OvHV-2 is responsible for sheep-associated malignant catarrhal fever (SA-MCF) disease in susceptible hosts, such as buffalo [3], cattle [4], deer [5], and rarely in pigs [4], and foals [6]. However, rabbits are susceptible to SA-MCF following intranasal nebulization of cell-free OvHV-2 and develop a disease similar to SA-MCF in terms of clinical signs and lesions [7, 8].

In OvHV-2, infection with non-adapted host species suggests latent infection and abortive lytic viral replication [9].

Typical SA-MCF, often referred to as the head-and-eye form, is the most common presentation of this condition in cattle [2]. Sick animals manifest pyrexia, anorexia, bilateral corneal opacity, nasal and ocular discharge, ulceration of various mucous membranes, and neurological manifestations in the terminal stages [10]. The most common gross pathological changes in SA-MCF-affected animals are erosions of the tracheal and bronchial mucosa, erythema of the turbinate mucosa, congestion, and edema of the lungs, and focal white lesions in the kidney [10]. Histopathologically, the hallmark of SA-MCF is lymphoproliferative inflammation with vasculitis involving medium-caliber arteries and veins, which is readily detected in most organs of cattle dying from acute SA-MCF [11]. Both clinical presentation and pathological features are of significant diagnostic value [12].

Detection of viral DNA by PCR in buffy coats and tissues, especially at high levels, can support the diagnosis of MCF-causing viruses (MCFV) [8]. Several serological assays have been used to detect antibodies against SA-MCF, but all assays use Alcelaphine herpesviruses as antigens, predominantly AlHV-1 because these viruses can be propagated in vitro [13]. These assays include virus serum neutralization (VN), immunoblotting (IFA), enzyme-linked immunosorbent assay (ELISA), competitive inhibition ELISA (cELISA), immunofluorescence assay (IF), immunoperoxidase assay (IPT), and drug complement fixation test [14–20]. However, the VN has very limited use in the detection of antibodies in animals infected with OvHV-2, and polyclonal antibody-based assays (ELISA, IFA, and IPT, among others) can detect antibodies against multiple epitopes of AlHV- 1 [21, 22]. To increase specificity, an ELISA has been developed using an Ov8 recombinant protein from OvHV-2 [23].

For the in vitro isolation of MCFV, primary cultures of bovine thyroid (BTh), bovine kidney (BK), bovine embryonic kidney (BEK), and calf testis (CT) cells have been used [22, 24, 25]. On the other hand, Hristov et al. (2016) described the use of primary rabbit kidney (RK) cell cultures and permanent cell cultures (Madin Darby bovine kidney (MDBK) cell line, embryonic bovine tracheal (EBTR) cell line, green monkey kidney cell line (VERO), and monkey cell line (MA-104)) for the isolation of the OvHV-2 virus. However, they mention that additional investigations are needed to confirm which type of OvHV-2 or AlHV-1 viral isolation they performed [26].

Cases of SA-MCF have been documented in North America, and economic losses to bison producers have been estimated at approximately US$1 million [27]. However, the direct economic losses associated with SA-MCF morbidity and mortality in Brazil were estimated at US$215,592 and US$214,368, respectively. If the data were projected nationally, it would result in a projected economic loss of US$3.5 to US$4.8 billion for the cattle industry in Brazil [28].

The first case of SA-MCF in Mexico was documented in Mexico City in a 6-year-old Holstein cow in 1969, which presented head and ocular clinical manifestations in addition to perivascular lymphoid infiltrate in various organs. However, the diagnosis was based only on a clinicopathological study [29]. More recently, an outbreak of SA-MCF occurred in the municipality of Tequisquiapan in the state of Queretaro, Mexico, affecting many animals that developed a typical head-and-eye clinical presentation.

Only one study has been carried out thus far investigating OvHV-2 in Mexico, as it is considered an exotic disease. Moreover, the economic and epidemiological impact of SA-MCF and the distribution and molecular characterization of the OvHV-2 virus in this country are unknown. Therefore, this study aimed to identify and characterize the OvHV-2 virus that causes the SA-MCF outbreak in horses and Artiodactyla kept at the Centro de Enseñanza, Investigación y Extensión en Producción Animal en Altiplano (CEIEPAA), Tequisquiapan, Queretaro, Mexico.

## Material and methods

### Ethics statements

All experimental procedures involving animals were conducted following the Institutional Subcommittee for the Care and Use of Experimental Animals of the Universidad Nacional Autónoma de México (UNAM) (SICUAE.DC-2020/3-5).

### Sampling location

**Fig 1:**
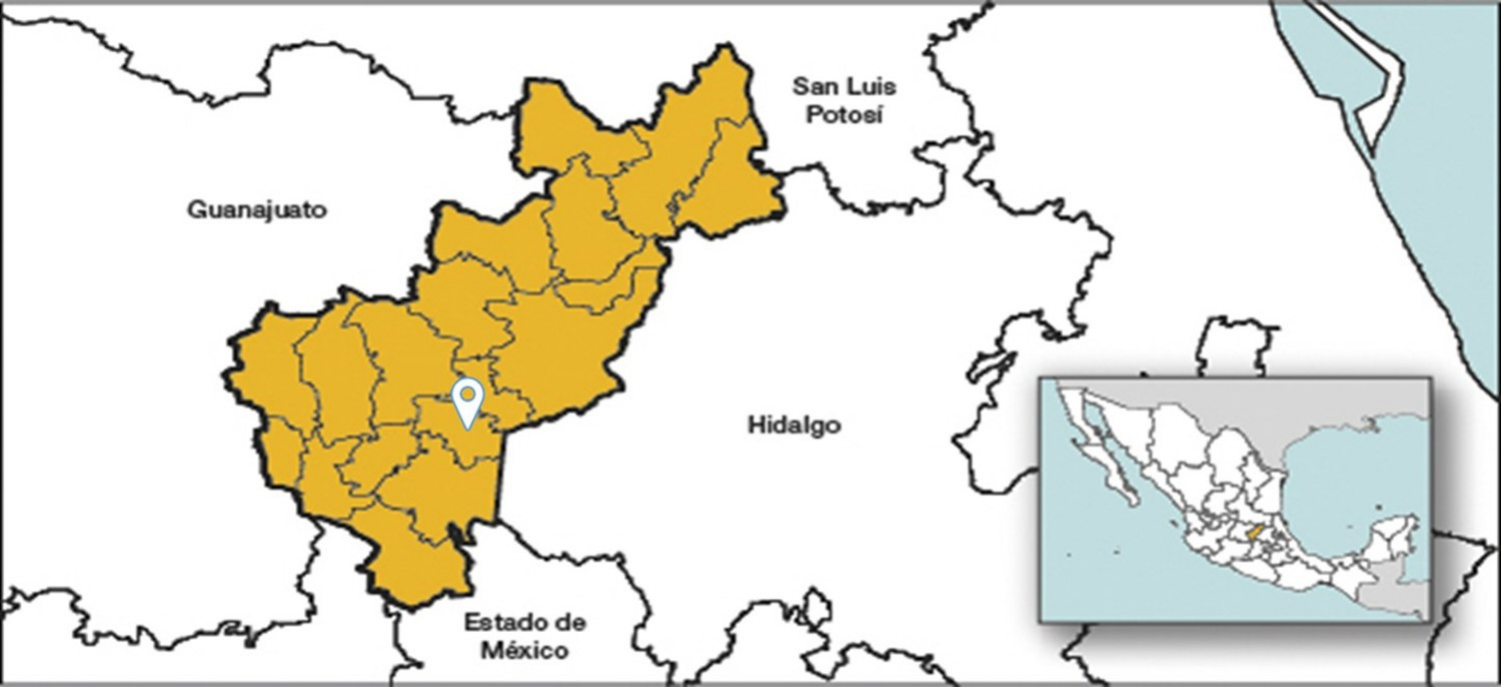
The geographical location of Tequisquiapan, Queretaro (Mexico), where the CEIEPAA is located (Modified from INEGI, 2023; https://www.inegi.org.mx/app/areasgeograficas/?ag=07000022#collapse-Mapas).

Sampling was performed at the CEIEPAA, belonging to the UNAM, located in Tequisquiapan, state of Queretaro, Mexico. This farm has an area of 186 ha. It is based on a semi-intensive production system with multiple species: deer (European red), dairy cows (Holstein, Jersey, and crosses), feedlot cattle (Limousin), sheep (Suffolk, Katahdin, and crosses), goats (Alpine Frances, Saanen, Toggenburg, and Boer) and horses (English thoroughbred, quarter horse, appendix, Portuguese, Santa Gertrudis, and Creole) (Fig 1). The species were separated between 1.14 and 2.8 km [30].

### The outbreak chronology

From August to December 2018, a vesicular disease occurred that affected all species of animals belonging to the CEIEPAA farm, and the United States-Mexico Commission for the Prevention of Foot-and-Mouth Disease and other Exotic Animal Diseases (CPA) took samples of serum, blood, epithelium of ulcerative lesions of the oral mucosa, deer organs (tongue, brain, submandibular lymph nodes, kidney, and spleen), and nasal swabs to carry out differential diagnoses of vesicular diseases [30]. In January 2019, UNAM conducted targeted sampling to identify and characterize the OvHV-2 virus.

### Supplementary tests

The serum samples were analyzed with ELISA tests to diagnose foot-and-mouth disease virus (FMDV) and vesicular stomatitis virus (VSV). On the other hand, samples of epithelium, blood, deer organs (tongue, brain, submandibular lymph nodes, kidney, and spleen), and nasal swabs were used to perform different molecular diagnoses: PCR or reverse transcription polymerase chain reaction (RT‒PCR) for FMDV, infectious bovine rhinotracheitis virus (IBRV), Indiana and New Jersey VSV, parapoxvirus (PRV), bluetongue virus (BTV), and contagious ecthyma virus (CEV). In addition to viral isolation of VSV (Table 1).

**Table 1:** Affected species, testing by PCR of other viruses and percentage of positive animals for OvHV-2.

Affected species	*Previous Diagnoses/Results (PCR)	**Clinical signs	Total, population/sick/dead	***Total number of animals sampled by CPA, OvHV-2 PCR positive, and percentage of positive	****Sampled animals, OvHV-2 PCR-positive and percentage of positive
**Horses**	VSV/ (-)	MUMV, UT, LFC, WL	39/14/0	35 [2] (5.71%)	39 [9] (23%)
**Feedlot cattle**	IBRV, VSV, PRV, FMDV, BTV/ (-)	MUMV, HS, LFC, WL	109/8/0	38 [8] (21.05%)	30 [11] (36%)
**Dairy cattle**	IBRV, VSV, PRV, FMDV, BTV/ (-)	MUMV, HS, LFC, WL	169/15/0	51 [6] (11.76%)	32 [5] (15%)
**Resident goats**	IBRV, VSV, PRV, FMDV, BTV/ (-)	-	271/0/0	30 [7] (23.33%)	32 [19] (59%)
**Goats in the quarantine area**	IBRV, VSV, PRV, FMDV, BTV, CEV/ (-)	MUMV	97/7/0	98 [35] (35.71%)	32 [8] (25%)
**Sheep**	IBRV, VSV, PRV, FMDV, BTV, CEV/ (-)	MUMV	246/37/0	67 [22] (32.83%)	30 [26] (86%)
**Deer**	IBRV, VSV, PRV, FMDV, BTV, CEV/ (-)	MUMV, HS, CO, LFC, WL	103/21/14	17 [8] (47.05%)	18 [8] (44%)
**Total**			**1034/65/14**	**336[88](26.19%)**	**213[86] (40.3%)**

Results of the nested end-point PCR for detecting the OvHV-2 virus in buffy coat samples of different species.

[] = positive to PCR, () = percentage of animals positive to PCR.

*Infectious bovine rhinotracheitis virus (IBRV), vesicular stomatitis virus serotypes Indiana and New Jersey (VSV), parapoxvirus (PRV), foot and mouth disease virus (FMDV), bluetongue virus (BTV), contagious ecthyma virus (CEV).

**mucogingival ulcers in the mouth vestibule (MUMV), ulcers in the tongue (UT), hypersalivation (HS), corneal opacity (CO), lower food consumption (LFC), and weight loss (WL).

*** Total number of animals sampled by CPA from August to December 2018

**** Total number of animals sampled by UNAM during January 2019

### Post-mortem evaluation

Of the 14 deer that died during the 2018 SA-MCF outbreak, 9 deer were assessed post-mortem because 5 were in an advanced state of decomposition. The results of the post-mortem evaluation were published by Pérez et al. (2022) [30]. Our current research presents the results of two of these deer (#172 and #174), which were chosen due to the severity of their clinical signs and lesions. Cornea, lymph nodes, tongue, rumen, liver, lung, and kidney samples were taken and fixed by immersion in 10% buffered formalin solution and routinely processed for paraffin embedding and sectioning using hematoxylin and eosin (H&E) staining for observation and histopathological analysis.

### Clinical sample collection and preparation

In January 2019, a new sampling was performed. Nevertheless, this time was aimed at animals that the CPA had previously tested positive for OvHV-2 by PCR, including those that still had ulcerative lesions (11 horses) and some healthy animals. The purpose of this sampling was to identify and characterize the OvHV-2 virus. Approximately 30 blood samples from each animal species were obtained in Vacutainer® tubes. Subsequently, the buffy coats were separated according to the methodology described by English & Andersen (1974) [31], and after they were stored at -196°C until processing for viral identification and PCR.

### Nested endpoint polymerase chain reaction (PCR) for OvHV-2 detection

Nested endpoint PCR was specific for the *ORF75* segment of OvHV-2, which encodes phosphoribosyl-formyl-glycinamidine synthase (FGARAT), which participates in purine metabolism and the production of viral tegument proteins [32].

Genetic material was extracted according to the manufacturer’s instructions for the High Pure PCR kit (Roche, Basel, Switzerland) [33]. Primer pairs designed by Baxter et al. (1993) were used, and nested PCR was performed according to the method described by Li et al. (1995), with some modifications [21, 32]. Briefly, in the first and second amplification reactions, 10 μM of each primer set was used, and the reaction volume was adjusted to 25 μL. A PCR Master Mix 5X (Taq-Load) was used. All reactions were performed using a GeneAmp® PCR System 9700 (Applied Biosystems, Waltham, MA, USA). A positive result generated a 238-bp band in the second amplification.

A bovine blood sample that tested positive for OvHV-2 by PCR and partial sequencing (ref. 6191), donated by CPA, was used as a positive control, and nuclease-free water was used as the negative control.

### Primary cell cultures

The primary cell culture was prepared using testes from 2-month-old male rabbits, which were humanely euthanized (SICUAE.DC-2020/3-5)]. Testes were washed five times with Hanks balanced salt solution (HBSS, Gibco, Life Technologies) supplemented with penicillin (100 U/mL, Gibco, Life Technologies), streptomycin (100 g/mL, Gibco, Life Technologies) and fungizone (2.5 g/mL, Gibco, Life Technologies). They were then placed in a sterile Petri dish where each testis’s tunica albuginea and connective tissue septa were dissected with sterile scissors. With the help of sterile forceps, manual cuts were made to obtain fragments smaller than 0.5 cm^2^ from both testes. The testes fragments were trypsinized for 1 h at 37°C under constant agitation using 0.25% trypsin-EDTA with phenol red (Gibco, Cat: 25200056) at a 1:5 ratio.

After incubation, the detached cells were filtered through a funnel with sterile gauze, and the filtrate was collected in a centrifuge tube and centrifuged at 800 × g for 10 min. The supernatant was discarded, and the pellet of cells was resuspended in Dulbecco’s modified Eagle’s medium (DMEM) supplemented with 10% fetal calf serum. Approximately 0.5 × 10^6^ cells/mL were seeded per bottle. Cultures were maintained in 25 cm^2^ flasks (Nunc, Roskilde, Denmark) at 37°C with a 5% CO_2_ atmosphere. Cells were monitored daily under an inverted Olympus microscope (IX71) with a 10× objective (Olympus Corporation, Shinjuku City, Tokyo, Japan), and after 2 days, the DMEM supplemented with 10% fetal bovine serum was changed to a similar fresh medium.

### Inoculation of primary cell cultures

For the identification of OvHV-2, 25-cm^2^ bottles with 80% cell confluence were used. Each bottle was inoculated with 200 μL of buffy coat lysate (PCR positive for OvHV-2). Lysates were obtained by freezing and thawing the buffy coats from each of the six species naturally infected with OvHV-2 (deer, dairy cattle, feedlot cattle, sheep, goats, and horses). Inoculated cells were propagated in DMEM supplemented with 5% fetal calf serum and incubated at 37°C with a 5% CO_2_ atmosphere. The flasks with the inoculated cells were observed every 24 h for 7 days using an Olympus inverted microscope (IX71) with a 10× objective (Olympus Corporation) to identify the specific cytopathic effect of OvHV-2 [26].

At least four blind passages were performed in all species except horses, in which fourteen blind passages were needed for virus adaptation to cell culture and cell-free virus production. Bottles with inoculated cells were frozen and thawed when cytopathic changes were observed in the monolayers (refractile cytomegalic cell foci, syncytia, or 50% destruction of the monolayer). Then, the culture supernatant was centrifuged at 1200 × g for 10 min, and the final supernatant was divided into small aliquots and stored at -80°C until use. For consecutive passages, fresh monolayers were prepared and inoculated with the virus supernatant mentioned above. When cytopathic changes were observed in the monolayers, the bottles with the inoculated cells were processed as mentioned above. Following the cytopathic changes of the monolayers, culture supernatants from the four blind passages were identified for the *ORF75* segment of the OvHV-2 virus DNA by nested PCR. As a negative control, a 25-cm^2^ bottle with 80% cell confluence was inoculated with 200 μL of buffy coat lysate from OvHV-2-negative horses.

The virus titer was quantified from the cell-free supernatants of the 4^th^ blind passage of all the species and from the 3^rd^ to the 14^th^ blind passages of the horse. Flat-bottomed 96-well plates were used as described by Reed and Muench [34]. To analyze the plates under a microscope, they were fixed 72 h post-infection with 4% paraformaldehyde diluted with distilled water and stained with 0.5% crystal violet diluted in 96° alcohol.

To observe intranuclear inclusion bodies (IBs), a 24-well plate at 80% cell confluence was inoculated with 200 μL of each of the four blind passages described above. The inoculated cells were propagated with DMEM supplemented with 2% fetal calf serum and incubated at 37°C for 24 h in a 5% CO_2_ atmosphere. Subsequently, the supernatant was removed, and the cells were fixed with 4% paraformaldehyde. Finally, hematoxylin stain was added for 1 min, washed with distilled water, and stained with eosin stain for 30 s. The bottom of each well was covered with glycerol and a coverslip. The intranuclear IBs were observed under an Olympus inverted microscope (IX71) with a 10× objective (Olympus Corporation). As a negative control, a 4-well plate was left at 80% cell confluence and inoculated with 200 μL of the buffy coat lysate from OvHV-2-negative horses; otherwise, the remaining analytical procedure was identical to that given above.

### Viral replication kinetics

The kinetics of viral replication were analyzed from the cell-free supernatants of the 4^th^, 5^th^, and 14^th^ passages of the buffy coat lysate from horses. Primary cultures of rabbit testis grown in 96-well flat bottom plates were inoculated and processed according to the technique of Reed and Muench [34]. Briefly, primary cultures were inoculated with serial 10-fold dilutions of each of the samples in quadruplicate per dilution. The inoculum was then removed at different time points (24, 48, 72, 96, and 120 h post-infection), and the plates were fixed with 4% paraformaldehyde for 10 min and stained with 0.5% crystal violet. Graphics were prepared using GraphPad Prism 9.5.1 software (GraphPad, USA).

### Partial sequencing of the ORF75 gene

Partial sequencing of the *ORF75* gene was performed from the fourth blind passage obtained by inoculating primary cultures with buffy coat lysate from different animal species (deer, dairy cattle, feedlot cattle, sheep, goats, and horses). Furthermore, partial sequencing of the CPA-donated positive control was performed. The seven samples were amplified by endpoint PCR to obtain a 422-bp fragment corresponding to a segment of the *ORF75* gene, and 300 ng of each PCR product was sequenced by bidirectional capillary electrophoresis through Sanger sequencing using external PCR primers as sequencing primers (MCF 556 and MCF 755) [32]. Electropherograms from each pair of sequencing reactions were edited and assembled with the Biological Sequence Alignment Editor (BioEdit), v 7.2.0; [35]. Sequences were aligned using MEGA 11 software [36].

### Phylogenetic analysis

The seven consensus sequences obtained using BioEdit software were compared with partial sequences of the *ORF75* gene reported in GenBank [35]. For the phylogenetic analysis, the alleles of high and low similarity for *ORF75* were selected using the BLAST (Basic Local Alignment Search Tool) of the NCBI (National Center for Biotechnology) [37].

Finally, the seven sequences were phylogenetically analyzed with sequences from the genus Macavirus (OvHV-2, AlHV-1, and AlHV-2) and human gammaherpesvirus types 4 and 8 (HHV-4 and HVH-8). The substitution model was selected with Jmodeltest, indicating that the Tamura-Nei model was the most appropriate for this data set [38, 39]. The analysis was performed with MEGA 11 software using the maximum likelihood method with 1,000 replicas [36].

The seven consensus nucleotide sequences of the *ORF75* gene fragment amplified in this work have been submitted to the GenBank public database and have been assigned the following accession numbers: ON375578 (horses), ON375579 (goats), ON375580 (deer), ON375581 (dairy cattle), ON375582 (feedlot cattle), ON375583 (sheep) and ON375584 (CPA positive control).

### Hyperimmune serum

To prepare an anti-OvHV-2 hyperimmune serum, we followed a protocol from a previous study by Orós et al. (1997), with some modifications. Three 9-week-old New Zealand white rabbits weighing approximately 1.7 kg and negative for OvHV-2 by nested endpoint PCR were used [40].

Rabbits were inoculated intramuscularly with the cell-free supernatant from the fourth blind passage of the horses (Tissue Culture Infectious Dose 10^4.19^ TCID_50_/mL) dissolved in phosphate-buffered saline (PBS) and a 2% Alhydrogel® (w/v) aqueous suspension of aluminum hydroxide gel adjuvant (InvivoGen). The supernatant from the previously mentioned fourth blind passage was inactivated with 0.2% formaldehyde diluted in distilled water.

Rabbits were housed in stainless-steel cages with wire mesh, fed commercial pellets, and had free access to running water. The animals were kept at a temperature of 23–25°C. After an acclimatization period, the rabbits received weekly intramuscular inoculation for 5 weeks. Subsequently, the rabbits were tranquilized (acepromazine 1 mg/kg), and their hearts were bled and then euthanized with an overdose of pentobarbital. The serum obtained was stored at -20°C until its subsequent use in immunofluorescence (IF), immunocytochemistry (ICC), and immunohistochemistry (IHC) techniques.

### Immunocytochemistry (ICC) and immunofluorescence (IF) techniques

To perform the ICC and indirect IF analyses, we modified the methodology described by Rossiter (1981). Two 24-well plates were used with a cell confluence of 80%, and the wells were inoculated with 200 μL of each of the four blind passages obtained previously [14].

Subsequently, they were incubated at 37°C for 72 h and fixed with 4% paraformaldehyde diluted in distilled water for 10 min at room temperature. The cells were permeabilized with 0.2% Triton X-100 for 5 min at room temperature. The plates were then washed three times with PBS. For ICC, endogenous peroxidase was inhibited by incubation with 3% hydrogen peroxide diluted in distilled water for 15 min and washed three times with distilled water. To reduce background noise, the two plates were incubated with 2.5% bovine serum albumin diluted in Tris-HCl buffer for 30 min at 37°C. Next, the two plates were incubated with anti-OvHV-2 rabbit hyperimmune serum diluted 1:5 in 0.1% albumin for 24 h at 4°C. After three washes with PBS, peroxidase-conjugated protein A was added to the ICC plate diluted 1:16 in 0.1% albumin and incubated for 1 h at 37°C, followed by three washes with PBS. The chromogen (hydrogen peroxide and 3,3´-diaminobenzidine) was added for 5 min, and the reaction was stopped with distilled water. Cells with positive immunoreactivity to the anti-OvHV-2 hyperimmune serum presented an insoluble brown coloration. The plate used for IF was processed in the same way as for ICC; however, endogenous peroxidase inhibition was omitted, and a fluorescein isothiocyanate (FITC)-conjugated anti-rabbit conjugate (anti-rabbit IgG) diluted 1:20 in 0.1% albumin was used as the conjugate. Cells with positive immunoreactivity to the anti-OvHV-2 hyperimmune serum were determined as those that presented an apple-green fluorescent emission at 520 nm. Immunoreactivity of the plates was observed using an Olympus epifluorescence inverted microscope (IX71) (Olympus Corporation). Uninoculated primary cell cultures and sera from rabbits negative for SA-MCF were used as negative controls.

### Immunohistochemistry

The specificity of the anti-OvHV-2 hyperimmune serum was evaluated in the liver of deer that died naturally during the SA-MCF outbreak at the CEIEPAA farm. This technique was performed as described by Headley et al. (2022), with some modifications [41]. Liver sections were mounted on positively charged slides (Biocare^TM^), deparaffinized with xylene, rehydrated with 0.1 M Tris-HCl buffer (pH 7.2), and transferred to a glass Coplin staining bottle with citrate buffer (0.1 M [pH 6.0]) for antigen retrieval. The procedure was performed in a pressure cooker for 10 min at 200°C. The slides were then left for 15 min at room temperature (RT) in a Coplin jar containing 0.1 M Tris-HCl buffer (pH 7.2). Subsequently, endogenous peroxidase was inhibited using 3% hydrogen peroxide diluted in distilled water. To reduce non-specific background staining, slides were incubated for 1 h at 37°C in a solution containing 0.1 M Tris-HCl buffer (pH 7.2), 1% dry milk, and 0.01% Triton X-100. This solution was decanted, and the slides were incubated overnight at 4% with rabbit anti-OvHV-2 hyperimmune serum diluted 1:5 in 0.1 M Tris-HCl buffer (pH 7.2) with bovine serum albumin (BSA) at 0.1%. After three washes with 0.1 M Tris-HCl buffer (pH 7.2), the slides were incubated with a peroxidase-coupled anti-rabbit IgG diluted 1:50 in 0.1 M Tris-HCl buffer (pH 7.2) with 0.1% BSA for 1 hour at RT. After three washes with 0.1 M Tris-HCl buffer (pH 7.2), a mixture of 3% hydrogen peroxide and 3,3’-diaminobenzidine was added to the slides to reveal the binding of anti-OvHV-2 hyperimmune serum in deer hepatocytes. Finally, the slides were counterstained with hematoxylin for 1 min. Positive immunoreactivity to anti-OvHV-2 hyperimmune serum was observed as an insoluble brown stain in the cytoplasm of the hepatocytes. A deer liver sample slide was used and processed as described above as a negative control, but anti-OvHV-2 rabbit hyperimmune serum was omitted.

## Results

### The outbreak chronology

On August 29, 2018, the first case of SA-MCF was suspected during the necropsy of a six-month-old goat belonging to the quarantine area that had recently been donated to the farm. The animal showed ulcerative lesions on the tongue and larynx; in addition, vasculitis and perivasculitis in different tissues were observed histopathologically. In September, 6 goats from the quarantine area and 15 Holstein cows had ulcerative lesions on the oral mucosa. SA-MCF is considered an exotic disease with mandatory reporting in Mexico, so on September 2, 2018, the outbreak was reported to the CPA. On September 3, the CPA first sampled the animals with and without clinical signs of all animal species to confirm the diagnosis. One week later, the CPA released the results, and the goats and dairy cows were confirmed to be negative for FMDV and VSV by ELISA and PCR. However, on October 4, 2018, six of the Holstein cows were PCR positive for OvHV-2 and negative for IBRV, PRV, and BTV.

By November, the disease had already spread to deer, feedlot cattle, and sheep. At that time, 21 deer had ulcerative lesions in the oral mucosa, and 5 blood samples and 3 tissues of these animals were PCR positive for OvHV-2 and negative for IBRV, VSV, PRV, FMDV, and CEV. On the other hand, 8 Limousine cattle had ulcerative lesions and were also positive for OvHV-2 by PCR. Finally, 10 sheep showed lesions suggestive of MCF, and on November 21, 2018, they were confirmed as positive for OvHV-2 by PCR.

In December 2018, 27 sheep had slight ulcerative oral cavity lesions and at the end of this month, the virus spread to horses; 14 of them displayed ulcerative lesions on the lips although only 2 were PCR positive for OvHV-2 and negative for VSV by RT‒PCR and viral isolation (Table 1). Most of the species recovered in a period of 20 days. However, the disease extended in deer for a few more days due to the severity of the ulcerative lesions in the oral mucosa, which made it impossible for them to eat.

During the outbreak, all animals of the farm received medical treatment based on immunological stimulants, vitamins A, D, and E, and the drinking water was alkalized. In November 2019 and 2020, there were two new outbreaks. Affected animals showed clinical signs similar to those observed in 2018. However, in these two new outbreaks, the most affected species was the horse due to the severity of the ulcers. In addition, in 2020, parturient goats also had ulcerative lesions on the oral mucosa.

### Post-mortem evaluation

**Fig 2:**
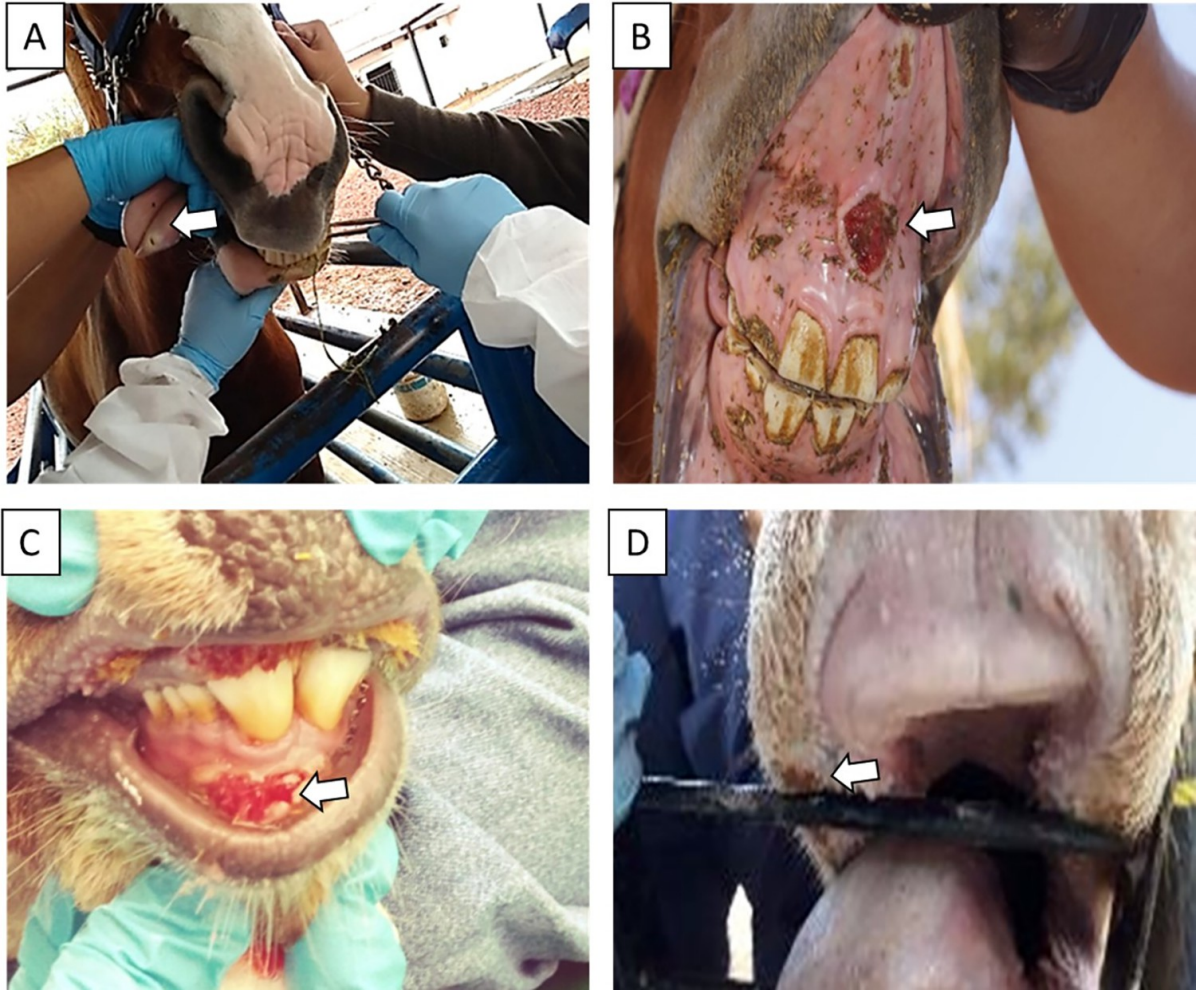
Ulcerative lesions in the oral mucosa of animals that survived the 2018 SA-MCF outbreak and deer that died 5 days after disease onset in CEIEPAA, Tequisquiapan, Queretaro, Mexico. (A) Horse with an ulcer on the apex of the tongue, (B) horse with an ulcer on the mucosa of the gums, (C) deer with ulcers on the mucosa of the upper and lower gums, (D) dairy cattle with an ulcer on the corner of the mouth. White arrows point to ulcers.

**Fig 3:**
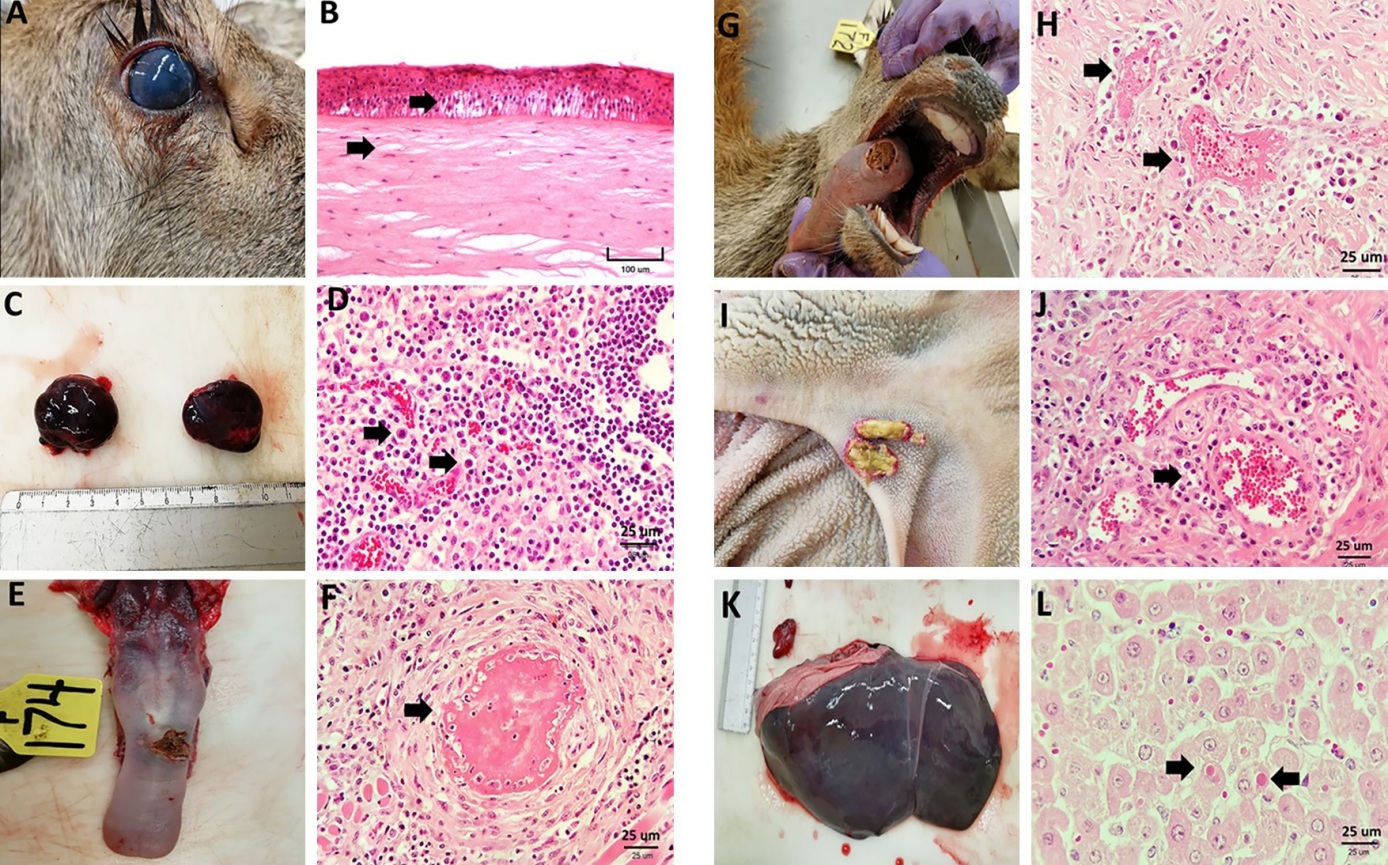
Post-mortem lesions were observed in deer #172 and #174 that died during the 2018 SA-MCF outbreak in CEIEPAA, Tequisquiapan, Queretaro, Mexico. (A) Corneal opacity, (B) cornea with edema and detachment of the corneal epithelium (black arrows), (C) enlarged, congested, and edematous retromandibular lymph nodes, (D) parafollicular region with numerous lymphocytes, plasma cells, and lymphoblasts (black arrows), (E) tongue with loss of epithelium continuity (ulcer) in the dorsal portion, (F) tongue with vasculitis and fibrin thrombus that occludes the lumen and fibrinoid necrosis (black arrow), (G) tongue with an ulcer in the dorsal portion of the tongue, with food encrustation, (H) tongue with thrombosis and infiltrate lymphoplasmacytic and lymphoblastic perivascular inflammation (black arrows), (I) rumen with focal ulcers in the pillar covered by a pseudomembrane, (J) rumen with perivascular inflammation composed of lymphocytes, plasma cells and lymphoblasts (black arrow), (K) enlarged and congested liver (hepatomegaly), (L) liver with areas of necrosis and cytoplasmic eosinophilic IB (black arrows).

The animals affected during the outbreak at CEIEPAA showed mucogingival ulcers in the buccal vestibule and tongue, hypersalivation, lower food consumption, weight loss, and corneal opacity (Fig 2). The two deer (#172 and #174) had marked retromandibular lymphadenomegaly, congested and edematous tongue, with ulcers on its dorsal part and the base. In addition, focal ulcers were observed in the rumen pillars, covered by a fibrinous pseudomembrane. The liver was slightly enlarged and congested. The main microscopic lesions observed were vasculitis and perivasculitis with fibrinous necrosis of blood vessels in the tongue and rumen, epithelial erosion, edema, and neovascularization of the cornea. The retromandibular lymph nodes showed lymphoid hyperplasia. The liver showed multifocal hepatic necrosis and cytoplasmic eosinophilic IB (Fig 3). No post-mortem lesions were observed in the kidney or lung.

### Buffy coat samples

A total of 213 buffy coat samples from the animals with clinical signs compatible with SA-MCF were tested by nested end-point PCR; 86 were positive (40.38%), and 127 were negative (59.62%). The PCR results revealed that resident goats and sheep had the highest percentage of animals positive for OvHV-2, whereas dairy cattle and horses had the lowest number of positive animals (Table 1).

### Inoculation of primary cell cultures

**Fig 4:**
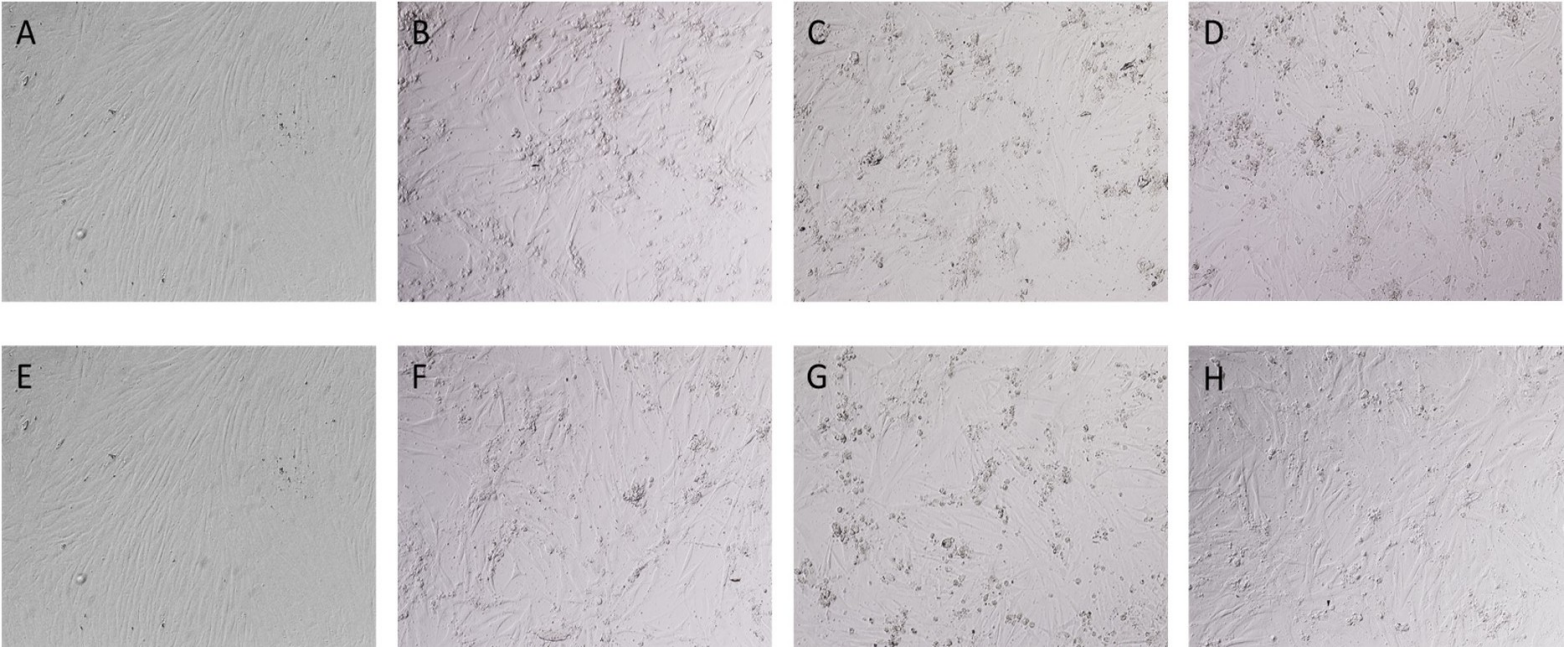
Cytopathic effect in primary cell cultures of rabbit testis cells inoculated with buffy coats of the different animal species affected during the SA-MCF outbreak at 72 h post-infection. (A) Negative controls, (B) fourth blind passage from horses, (C) fourth blind passage from sheep, (D) fourth blind passage from sheep, (E) negative control, (F) fourth blind passage from deer, (G) fourth blind passage from dairy cattle, (H) fourth blind passage from feedlot cattle. Magnification: 100X.

The cytopathic effect (CPE) caused by the buffy coat lysate pools, characterized by changes in primary culture morphology, generated small clusters of refractile cytomegalic cells between 48 and 72 h post-infection and was similarly induced with the six buffy coat lysate pools used (Fig 4).

Buffy coat lysates from sheep and goats induced marked CPE with 50% destruction of the monolayer from the first passage to 72 h and 100% between 96 and 120 h post-infection. Similarly, the percentage of CPE increased from the second passage and fourth passage onward for goat and sheep buffy coat lysates, respectively (Table 2). In contrast, primary cell cultures inoculated with buffy coat lysate pools from horses, dairy cattle, feedlot cattle, and deer showed low CPE, ranging between 20 and 30% in the first and second passages, respectively. In contrast, in the third and fourth passages, monolayer destruction increased to 50% and 90% at 72 h post-infection (Table 2).

**Table 2:** Cytopathic effect in primary cell cultures.

Affected species	CPE*	CPE*	CPE*	CPE*
	1° passage	2° passage	3° passage	4° passage
**Horses**	20–30%	20–30%	50%	90–100%
**Feedlot cattle**	20–30%	20–30%	20–30%	90–100%
**Dairy cattle**	20–30%	20–30%	90–100%	90–100%
**Goats**	50%	90–100%	90–100%	90–100%
**Sheep**	50%	20–30%	20–30%	90–100%
**Deer**	20–30%	20–30%	50%	90–100%

Percentage of cytopathic effect in primary cell cultures of rabbit testis.

CPE* = Cytopathic effect (percentages reflect the degree of cellular monolayer destruction

In cell cultures, viral titers obtained from the cell-free supernatants of the 4^th^ blind passage at 72 h post-infection were as follows: horses 10^4.19^ TCID_50_/mL, goats 10^3.8^ TCID_50_/mL, deer 10^3.3^ TCID_50_/mL, dairy cattle 10^3.5^ TCID_50_/mL, feedlot cattle 10^2.8^ TCID_50_/mL and sheep 10^3.5^ TCID_50_/mL.

**Fig 5:**
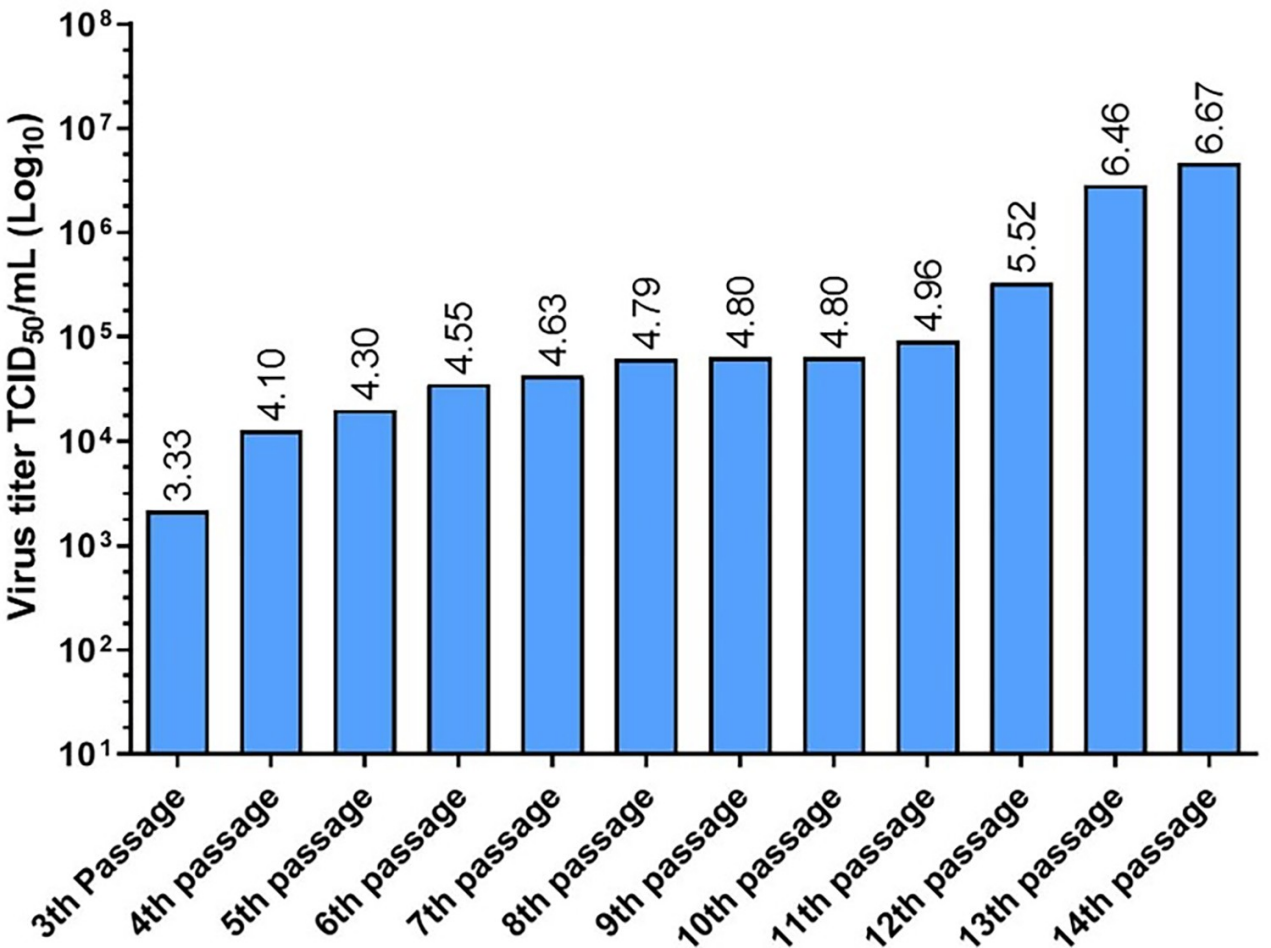
Virus titration of the supernatants from the 3^rd^ to 14^th^ passages of the buffy coat lysate from horses. Continuous passage of the buffy coat lysate from horses in primary rabbit testis cultures led to an increase in viral titer.

Viral titers of the cell-free supernatants of the 3^rd^ to 14^th^ passages from the buffy coat lysate from horses were collected at 72 h post-infection in each passage, and the CPE in crystal violet-stained wells was read (Fig 5). We determined that the viral titer of the 3^rd^ passage was 10^3.3^ TCID_50_/mL, while the viral titers of passages 4, 5, 6, 7, 8, 9, 10, and 11 ranged from 10^4^ TCID_50_/mL to 10^4.96^ TCID_50_/mL, which were very similar. However, an increase in titers was observed in passage 12, 13, and 14 viruses with titers of 10^5.52^ TCID_50_/mL, 10^6.46^ TCID_50_/mL, and 10^6.67^ TCID_50_/mL, respectively.

**Fig 6:**
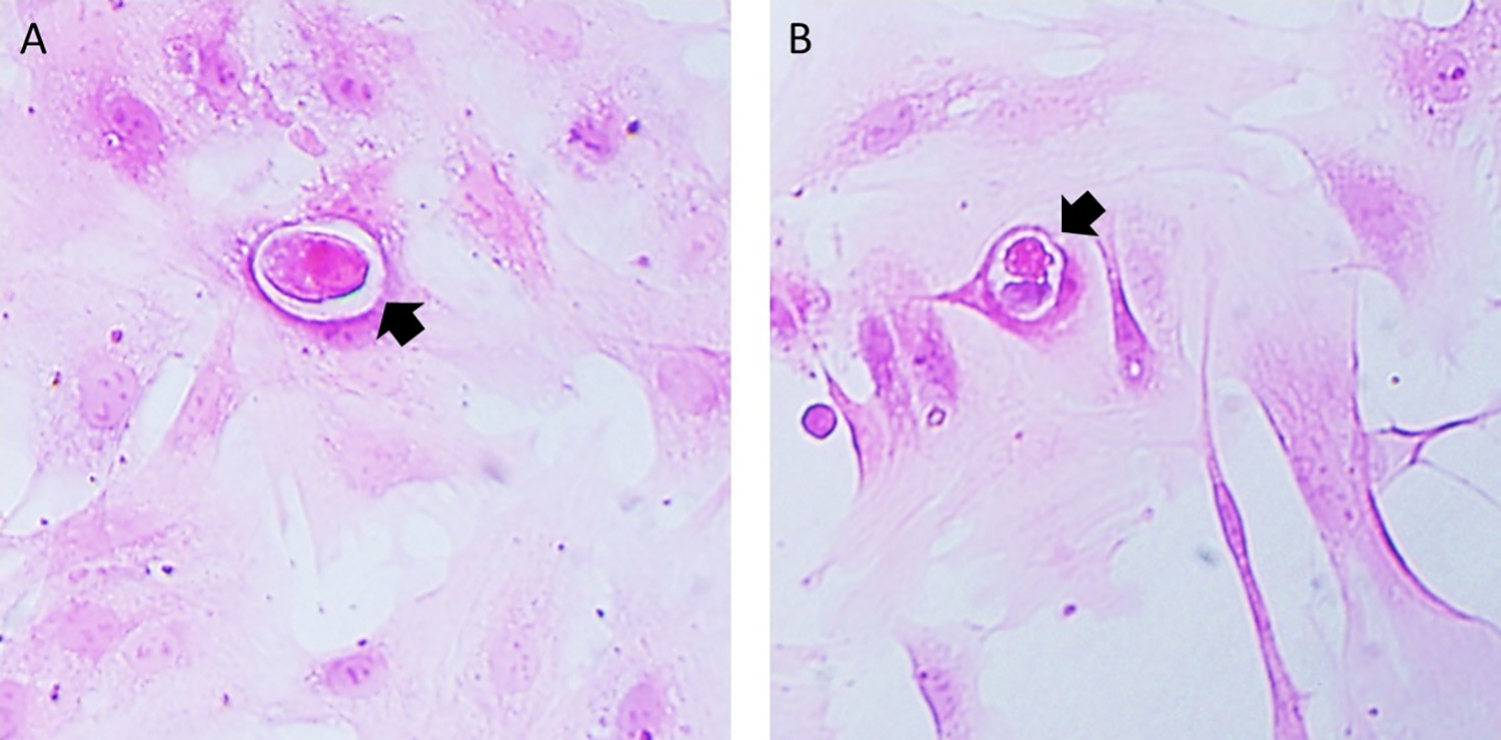
Intranuclear acidophilic IBs were observed at 24 h post-infection. **(**A) Fourth blind passage from horses, (B) fourth blind passage from goats. The black arrows indicate the intranuclear IB. Magnification: 200X. H&E staining.

Intranuclear acidophilic IBs were observed in primary cell cultures of rabbit testis cells inoculated with the cell-free supernatants of the four blind passages at 24 h post-infection. However, in the wells containing the cells inoculated with cell-free supernatants of the fourth blind passages from goats and horses, more than five IBs were observed (Fig 6). In contrast, in wells inoculated with cell-free supernatants of the fourth blind passages from dairy cattle, feedlot cattle, sheep, and deer, fewer than three IBs were observed in the entire well.

### Nested end-point PCR for OvHV-2 detection

**Fig 7:**
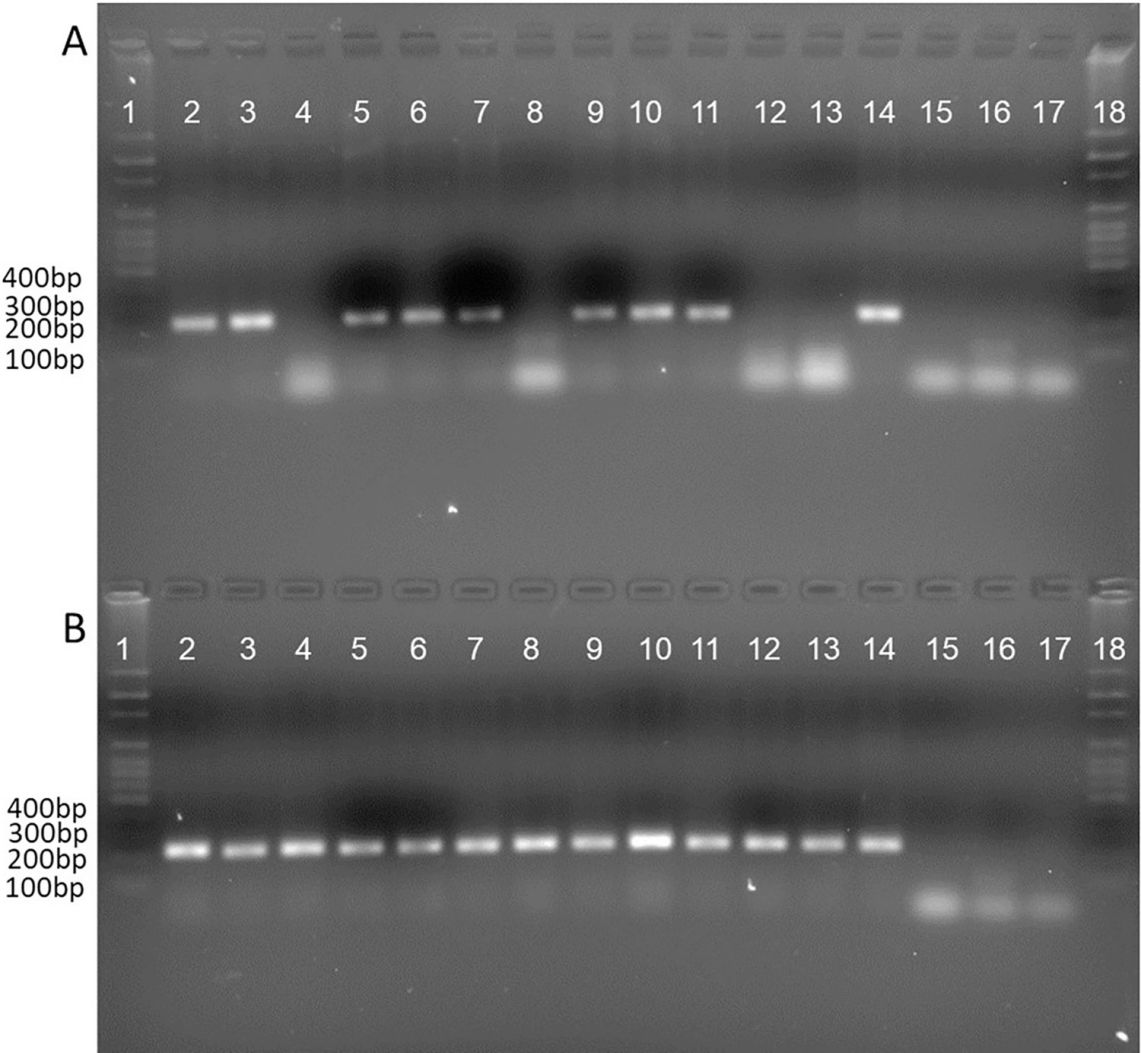
Agarose gel (1.5%) run in TAE 1X buffer. (A) Nested PCR of the first and second passages of primary cell cultures of rabbit testis. (1) Molecular weight marker 1 kb, (2) first blind passage from horses, (3) second blind passage from horses, (4) first blind passage from sheep, (5) second blind passage from sheep, (6) first blind passage from goats, (7) second blind passage from goats, (8) first blind passage from deer, (9) second blind passage from deer, (10) first blind passage from dairy cattle, (11) second blind passage from dairy cattle, (12) first blind passage from feedlot cattle, (13) second blind passage from feedlot cattle, (14) positive control, (15) negative control of amplification (NCA), (16) negative control of extraction (NCE), (17) non-inoculated primary cell cultures (NPCC), (18) molecular weight marker 1 kb. (B) Nested PCR of passages three and four in primary cell cultures of rabbit testis. (1) Marker of molecular weight of 100 bp, (2) third blind passage from horses, (3) fourth blind passage from horses, (4) third blind passage from sheep, (5) fourth blind passage from sheep, (6) third blind passage from goats, (7) fourth blind passage from goats, (8) third blind passage from deer, (9) fourth blind passage from deer, (10) third blind passage from dairy cattle, (11) fourth blind passage from dairy cattle, (12) third blind passage from feedlot cattle, (13) fourth blind passage from feedlot cattle, (14) positive control, (15) negative control of amplification (NCA), (16) negative control of extraction (CNE), (17) non-inoculated primary cell cultures (NPCC), (18) molecular weight marker 1 kb.

DNA was extracted from the cell-free supernatants of the four blind passages obtained from the infection of the primary cultures with buffy coat lysate pools from the different animal species affected during the SA-MCF outbreak. The cell-free supernatants from horses, goats, and dairy cattle were positive by PCR for OvHV-2 from the first passage. The cell-free supernatants from sheep and deer were positive until the second passage. However, the cell-free supernatant of feedlot cattle was negative by PCR until the third and fourth passages (Fig 7).

### Viral replication kinetics

**Fig 8:**
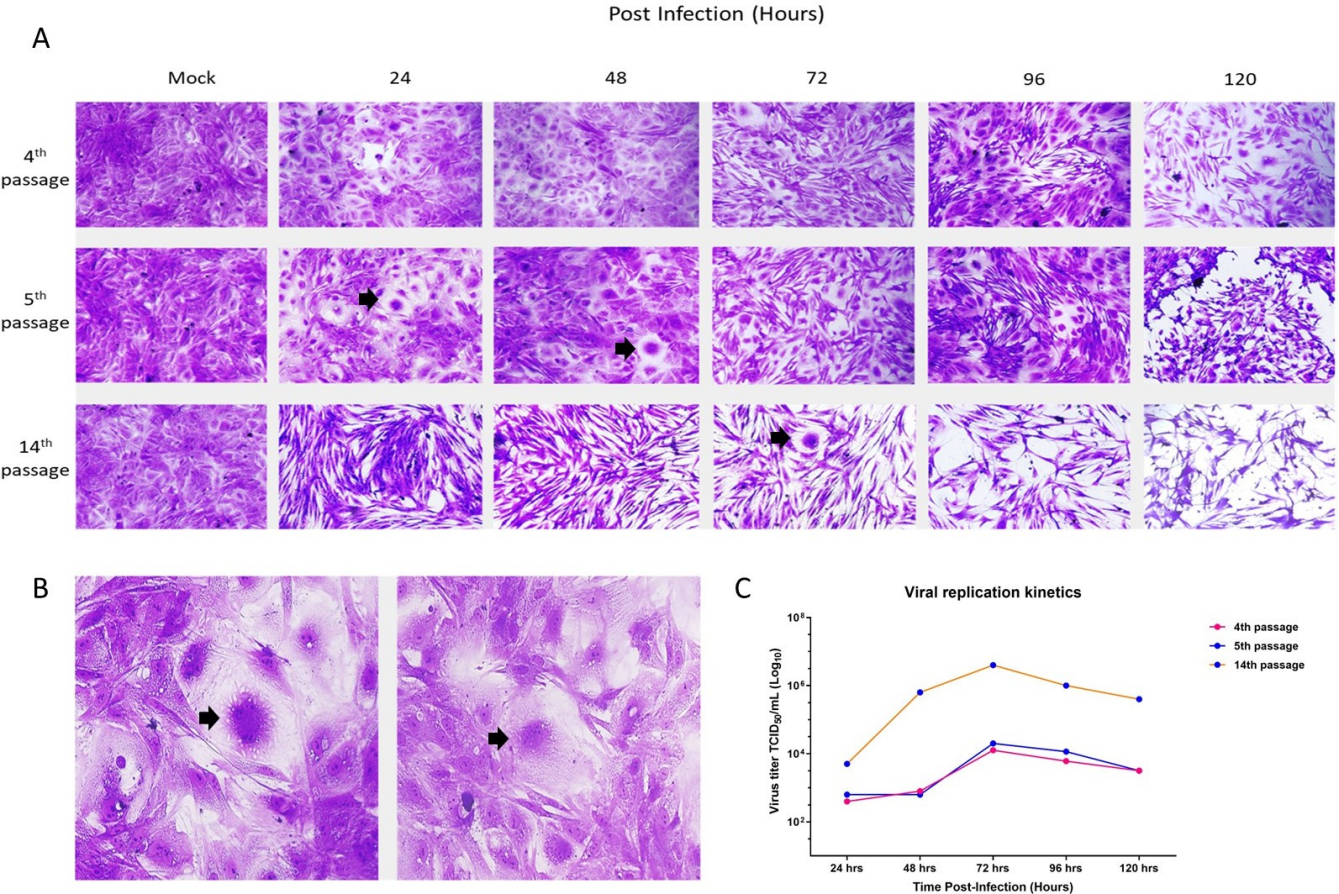
Growth kinetics of the cell-free supernatants from the 4^th^, 5^th,^ and 14^th^ passages of the buffy coat lysate from horses. (A) Cells inoculated with supernatant from the 4^th^ passage, upper panel; supernatant from the 5^th^ passage, middle panel; and supernatant from the 14^th^ passage, lower panel. Magnification: 100X. (B) Syncytia were observed in the 5^th^ passage at 24 h and 48 h post-infection (black arrows). Magnification: 200X. (C) Graph of viral titers at 24, 48, 72, 96, and 120 h post-infection corresponding to the 4^th^, 5^th^, and 14^th^ passages of the buffy coat lysate from horses, where a maximum replication peak is observed at 72 h post-infection.

The growth kinetics study showed that the buffy coat lysate from horses replicated rapidly, and syncytia could be observed starting at 24 h post-infection in the 5^th^ passage and up to 72 h in the 14^th^ passage (Fig 8A).

The 4^th^ and 5^th^ passages achieved similar peak titers of 10^4.1^ TCID_50_/mL and 10^4.3^ TCID_50_/mL at 72 h post-infection, respectively. However, the 14^th^ passage achieved higher peak titers of 10^6.6^ TCID_50_/mL at 72 h post-infection. In addition, at 120 h post-infection, the viral titers decreased (Fig 8C).

### Phylogenetic analysis

**Fig 9:**
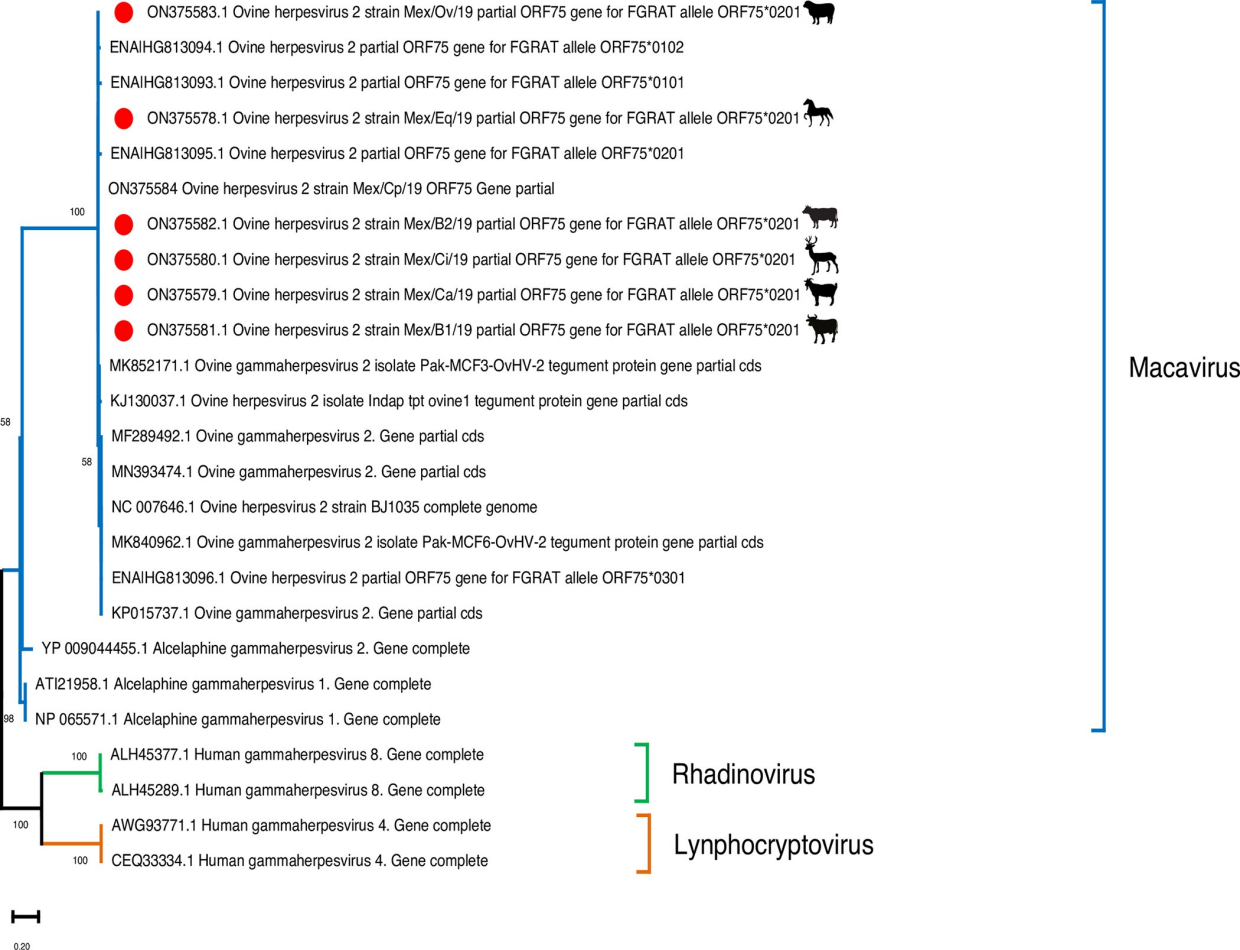
Phylogenetic analysis of partial sequences of the *ORF75* gene. The tree is at scale, and the bar indicates the number of substitutions per site. The Mexican sequences are marked with red circles.

The alignment of the partial sequences of 422 bp of the *ORF75* gene indicated that the six partial sequences from the cell-free supernatants of the fourth passage from the different species affected by SA-MCF and the positive control of OvHV-2 shared 98%-99% nucleotide identity with other partial *ORF75* sequences obtained from the GenBank database. Regarding the structure of the inferred topology, the analysis indicated that our seven partial sequences present high homology with macaviruses with different OvHV-2 *ORF75* genes, reported in different parts of the world and separated from the clade of rhadinoviruses (human gammaherpesvirus 8) and from lymphocryptovirus (human gammaherpesvirus 4), found in different clades (Fig 9).

On the other hand, the *ORF75* partial sequences of the six cell-free supernatants of the fourth passage compared to the BJ1035 sequence, presented a non-synonymous substitution at position 1213, changing the amino acid (AA) from lysine (K) to threonine (T) (Table 3); however, the partial sequence of the sheep presented a second mutation at position 1314, changing the AA cysteine (C) to glycine (G) (Table 3), and therefore, they were classified as ORF75*0201 due to the number of substitutions according to Russell’s classification et al. (2014).

**Table 3:** Genetic diversity of Mexican *ORF75* sequences compared to the reference gene BJ1035.

GENE *ORF75*	NT [3637]	NT [3638]	NT [3639]	AA [1213]	NT [3940]	NT [3941]	NT [3942]	AA [1314]
* **REF NC_007646.1 BJ1035**	A	A	A	LYS	T	G	C	CYS
**ON375578.1 (HORSE)**	A	C	G	**THR**	-	-	-	NA
**ON375579.1 (GOAT)**	A	C	G	**THR**	-	-	-	NA
**ON375580.1 (DEER)**	A	C	G	**THR**	-	-	-	NA
**ON375581.1 (DAIRY COW)**	A	C	G	**THR**	-	-	-	NA
**ON375582.1 (FEEDLOT CATTLE)**	A	C	G	**THR**	-	-	-	NA
**ON375583.1 (SHEEP)**	A	C	G	**THR**	G	G	C	**GLY**

*Ref = reference, () = source, NT = nucleotides, AA = amino acids, [] = position, NA = does not apply.

### Immunocytochemistry (ICC) and immunofluorescence (IF) techniques

**Fig 10:**
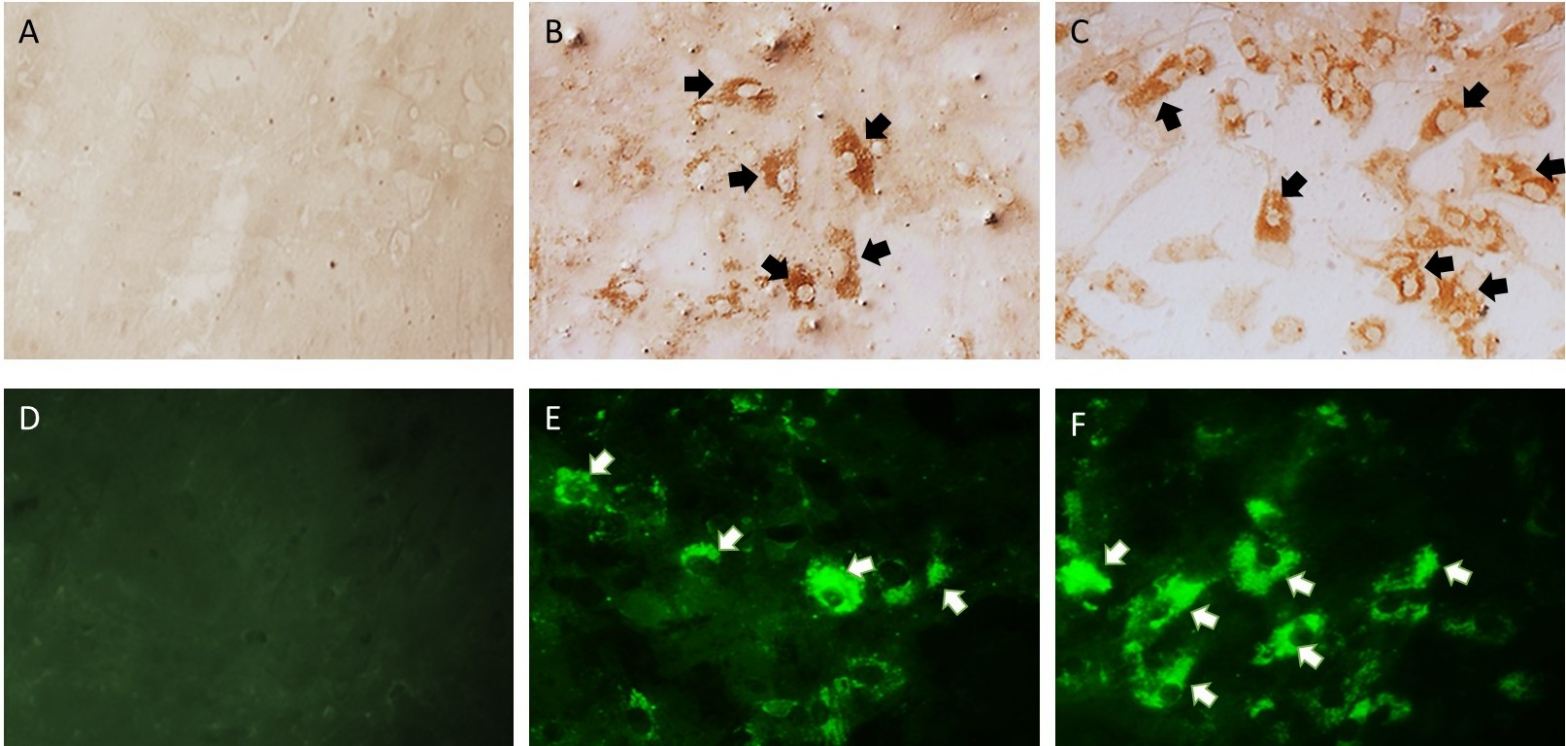
Cytoplasmic immunoreactivity with the ICC and IF techniques in primary cell cultures of rabbit testis cells. (A) ICC negative control, (B) ICC of the third passage from horses, (C) ICC of the third passage from goats, (D) IF negative control, (E) IF of the third passage from horses, (F) IF of the third passage from goats. Arrows indicate the cytoplasmic immunoreactivity appearing as an insoluble brown color for the ICC and a green-apple color for the IF. Magnification: 200X.

Positive cytoplasmic immunoreactivity to anti-OvHV-2 rabbit hyperimmune serum was observed in all wells inoculated with cell-free supernatants of the four blind passages from horses, goats, dairy cattle, feedlot cattle, sheep, and deer. However, with the third blind passage of horses and goats, a higher proportion of cells (40% and 50% of the monolayer) with positive immunoreactivity to rabbit serum was observed (Fig 10). In contrast, wells inoculated with the third and fourth passages of feedlot cattle, dairy cattle, sheep, and deer had a lower proportion of cells (10% of the monolayer), with cytoplasmic immunoreactivity. Apple-green emission at 520 nm by IF and a brown precipitate by ICC were evident.

### Deer liver immunohistochemistry

**Fig 11:**
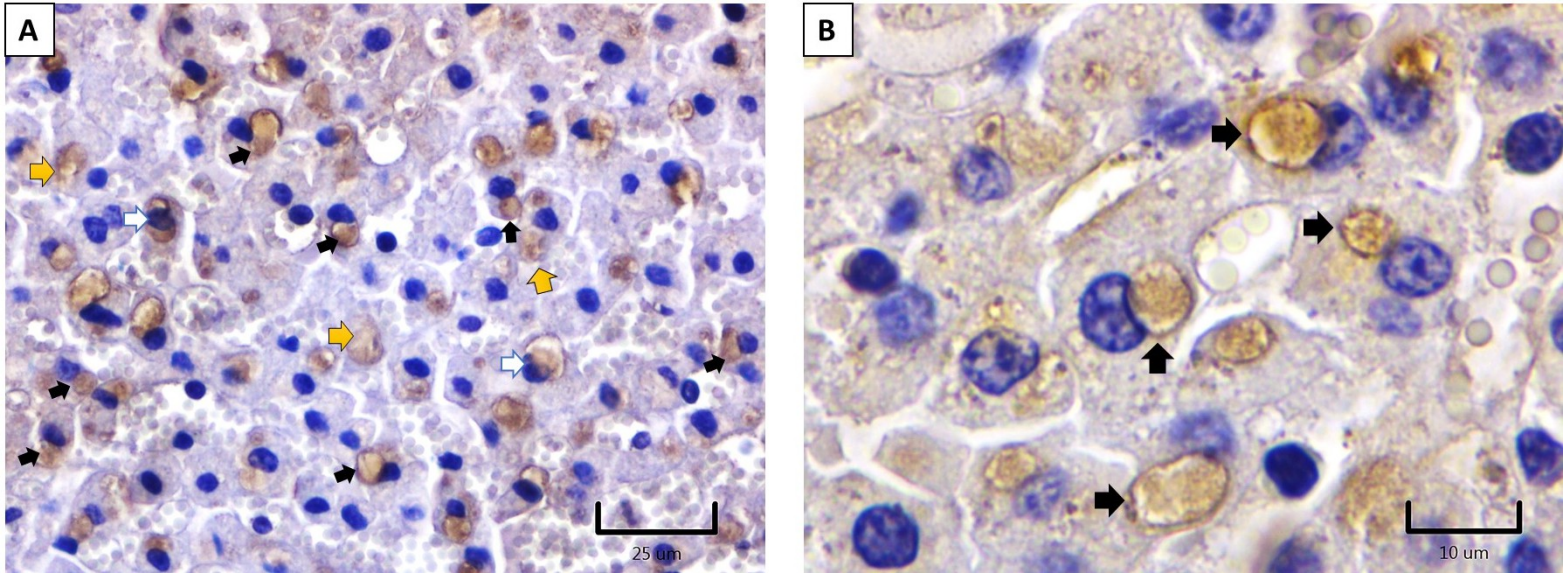
Deer liver immunohistochemistry. (A) and (B) Immunoreactivity to OvHV-2 antigens with rabbit hyperimmune serum in cytoplasmic IBs (black arrows), within the cytoplasm (yellow arrows), and in the nucleus of hepatocytes (white arrows).

The cytoplasmic IBs of hepatocytes from the affected deer were immunoreactive to anti-OvHV-2 hyperimmune rabbit serum. In addition, also slight intranuclear immunoreactivity was also observed in hepatocytes (Fig 11).

## Discussion

An outbreak of SA-MCF that occurred in CEIEPAA, located in Tequisquiapan, state of Querétaro, Mexico, is described in the present study. The etiologic agent was identified and characterized as OvHV-2 using different diagnostic techniques, including IF, ICC, CPE, end-point PCR, and partial sequencing of the *ORF75* gene. During the outbreak, many animals of different species were affected, showing typical head-and-eye clinical signs compatible with SA-MCF and corneal opacity [11]. The microscopic lesions of vasculitis and perivasculitis in different organs and varying degrees of thrombosis and lymphoid hyperplasia in lymph nodes observed in deer that died during the SA-MCF outbreak are consistent with those reported by Brown et al. (1992) in white-tailed deer [42].

This study used buffy coats from affected animals to identify that they were infected with the OvHV-2 virus. This approach is similar to that of a study by Hristov et al. (2016), who used bison buffy coats, whole blood, lung, and spleen samples from differently affected animals during an outbreak of SA-MCF [26].

Primary cultures of rabbit testis were used for the in vitro identification of OvHV-2. The use of primary cultures to perform viral identification had already been reported previously for AlHV-1 and AlHV-2 [22, 24, 25]. From the third passage on primary cell cultures of rabbit testis, a marked CPE was observed at 72 h post-infection, consisting of small foci of refractile cytomegalic cells. This CPE was also described by Rossiter et al. (1980) during the identification and isolation of AlHV-1 [43].

In the present study, titers of the fourth blind passages from horses, goats, sheep, deer, feedlot cattle, and dairy cattle obtained in primary cell cultures of rabbit testis ranged from log 10^2.8^ to log 10^4.19^ TCID_50_/mL. On the other hand, the cell-free supernatant from horses reached its highest peak of 10^6.6^ TCID_50_/mL in passage 14 at 72 h post-infection. These results indicated that propagation of the OvHV-2 virus in primary rabbit testis cultures led to an increase in viral titers at each passage. This viral titer is similar to that found by Hristov et al. (2016) of 10⁶ ТCID _50_/mL in Madin-Darby bovine kidney (MDBK) cells after passage 19 [26]. This increase in viral titers at higher passages could be because in the first passages, the virus is predominantly cell-associated, and after five passages, there is a period during which the viral genome undergoes rearrangements, leading to viral attenuation and resulting in better cell-free virus yields. However, this has only been shown in AlHV-1 [44]. In addition, in primary cultures of rabbit testis inoculated with buffy coats, we identified intranuclear acidophilic IB by H&E staining, which coincides with the results reported by Hristov et al. (2016) [26].

OvHV-2 was identified in primary cell cultures from rabbit testis through end-point nested PCR targeting a 238-bp fragment of the *ORF75* gene, with a greater intensity starting in the third and fourth passages in all cell-free supernatants from the different affected species. These results indicate that several blind passages are required to generate better cell-free virus yields and achieve adaptability of the virus to the cell, consistent with the report by Hristov et al. (2016) [26].

The buffy coat lysate from horses replicated with similar growth kinetics in the 4^th^ and 5^th^ passages, however, in the 14^th^ passage, it reached a higher titer. Viral replication could be detected starting at 24 h post-infection due to syncytes formation only in the 5^th^ passage and 14^th^ passage. The CPE gradually increased, reaching a maximum at 72 h post-infection and the decrease in the viral titer at 120 h post-infection could be due to the death of cells. With these results, we can infer that primary cultures of rabbit testis support OvHV-2 replication. Our results agree with those of Hristov et al. (2016), in that the bison buffy coat was characterized by small syncytia at 24 h post-infection, and between 60–72 h post-infection there was more than 50% destruction of the monolayer [26].

Furthermore, the definitive diagnosis of OvHV-2 infection was confirmed using partial sequencing of the 422-bp fragment of the *ORF75* gene. Interestingly, the seven sequences from the cell-free supernatants of the fourth passage from different species affected by SA-MCF and the positive control of OvHV-2 shared 98%-99% nucleotide identity among them and with the OvHV-2 sequences reported in different regions of the world. These results align with those reported by Martins et al. (2017), who sequenced 13 positive samples of bovines from different areas of Brazil and found 97%-100% similarity with OvHv-2 from several other regions of the world [45].

In the present study, the *ORF75* sequences were classified as ORF75*0201 for presenting at least one non-synonymous substitution, change from AA lysine to threonine; this indicates that the infectious agent that affected all animals during the 2018 outbreak with SA- MCF was the same strain as OvHV-2. On the other hand, our results differ from those of Russell et al. (2014) because they found that the major allele in 18 samples was ORF75*0101, which they found to be similar to the reference sequence BJ1035 [46, 47]. We can infer that in Mexico the most circulating allele is ORF75*0201. This could be a novel allele that allows infection between species that should be monitored. However, for genetic characterization and epidemiological studies, we would need to sequence the *ORF50* and *Ov9*.*5* genes, the latter being the most important for studies of this type [46]. In this sense, and with the results presented above, we could infer goats and sheep as sources of infection (transmission between species) or as potential reservoirs able to transmit to susceptible species (horses, deer, dairy cows, and feedlot cattle) due to their closeness to each other and sharing some of these grasslands.

The cytoplasmic immunoreactivity observed through IF and ICC in primary cell cultures from rabbit testis is equivalent to the intracytoplasmic immunoreactivity observed in multiple tissue samples affected by OvHV-2 using a monoclonal antibody (mAb-15A) targeted at a conserved epitope among all macaviruses [41]. In contrast, cytoplasmic acidophilic (eosinophilic) IBs immunoreactive to OvHV-2 using rabbit hyperimmune serum were observed in hepatocytes of deer that died during the SA-MCF outbreak. These results are consistent with those reported by Aluja et al. (1969), who described cytoplasmic acidophilic IBs in the neurons of cows infected with SA-MCF [29]. Similarly, our results are consistent with those reported by Goss et al. (1947), who described these IBs in Purkinje cells and epithelial cells of mucous membranes [48]. The cytoplasmic acidophilic IBs observed in the present study could be due to viral multiplication and the overproduction of viral and cellular proteins. These macromolecular aggregates, also called “dense bodies” in transmission electron microscopy, are very common in human cells inoculated with cytomegalovirus. Most of these structures are sequestered in Golgi-derived vesicles, and some dense bodies are located near or in contact with the nuclear envelope, where they appear to be transferred through nuclear pore viral proteins necessary for viral assembly [49]. The presence of acidophilic IB, as a manifestation of cytoplasmic accumulation of viral protein, also coincided with that described by Nelson et al. (2013), suggesting that cytoplasmic distribution of the capsid protein in OvHV-2 in infected terminal hosts represents abortive viral replication [9]. As a result, it can be speculated that cytoplasmic IBs in deer hepatocytes are formed by the accumulation of viral proteins necessary for virion assembly, in addition to abortive virions and cellular proteins. Further studies are needed to confirm this hypothesis.

## Conclusions

The SA-MCF outbreak that affected CEIEPAA animals in Tequisquiapan, Queretaro, Mexico, was caused by the OvHV-2 virus. In addition, it was shown for the first time in Mexico that horses are susceptible to OvHV-2 infection and develop SA-MCF, so it should be considered in the differential diagnosis of vesicular diseases in horses. Considering all results described above, we demonstrated that the buffy coats of CIEEPAA horses and Artiodactyla were infected with the OvHV-2 virus. Furthermore, we determined that primary rabbit testis cultures could be a good choice of cell culture model for OvHV-2 virus identification, as they support viral replication, CPE development, intranuclear IBs, and cytoplasmic immunoreactivity. However, to demonstrate the viral isolation of OvHV-2 in primary rabbit testis cultures, the disease needs to be reproduced in a susceptible host, through experimental inoculation with the isolate.

We also showed that the Mexican *ORF75* sequences shared 98%-99% nucleotide identity among them and with the OvHV-2 sequences reported in different regions of the world, and the ORF75*0201 allele affected all animals in the CEIEPAA during the 2018 SA-MCF outbreak.

## Supporting information

S1 Raw images (TIF)
