# Supplementary figures and images for: First identification and characterization of ovine gammaherpesvirus type 2 in horses and artiodactyla from an outbreak of malignant catarrhal fever in Mexico
Source: PLoS One. 2023 Sep 1;18(9):e0290309. doi: 10.1371/journal.pone.0290309 (PMC10473478; doi:10.1371/journal.pone.0290309)

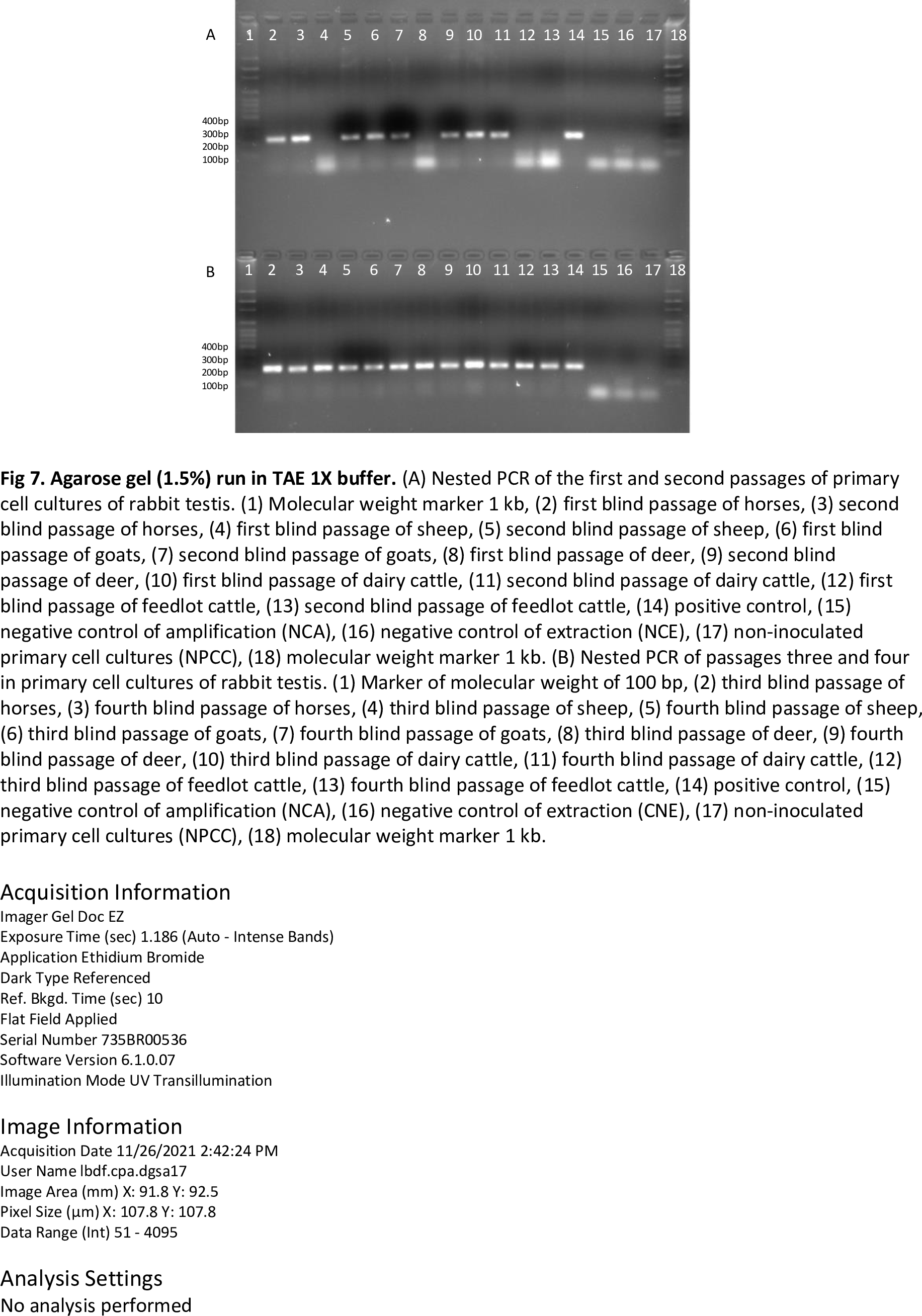

Supplement: S1 Raw images — (TIF) [file pone.0290309.s001.tif]
